# A Nomogram-Based Model to Predict Respiratory Dysfunction at 6 Months in Non-Critical COVID-19 Survivors

**DOI:** 10.3389/fmed.2022.781410

**Published:** 2022-02-23

**Authors:** Rebecca De Lorenzo, Cristiano Magnaghi, Elena Cinel, Giordano Vitali, Sabina Martinenghi, Mario G. Mazza, Luigi Nocera, Marta Cilla, Sarah Damanti, Nicola Compagnone, Marica Ferrante, Caterina Conte, Francesco Benedetti, Fabio Ciceri, Patrizia Rovere-Querini

**Affiliations:** ^1^Medical Residency Program, Vita-Salute San Raffaele University, Milan, Italy; ^2^Division of Immunology, Transplantation and Infectious Diseases, San Raffaele Hospital, Milan, Italy; ^3^Unit of Psychiatry and Clinical Psychobiology, Division of Neuroscience, San Raffaele Hospital, Milan, Italy; ^4^Unit of General Medicine and Advanced Care, San Raffaele Hospital, Milan, Italy

**Keywords:** COVID-19, long-term, respiratory sequelae, follow-up, prediction algorithm

## Abstract

**Objective:**

To assess the prevalence of respiratory sequelae of Coronavirus disease 2019 (COVID-19) survivors at 6 months after hospital discharge and develop a model to identify at-risk patients.

**Patients and Methods:**

In this prospective cohort study, hospitalized, non-critical COVID-19 patients evaluated at 6-month follow-up between 26 August, 2020 and 16 December, 2020 were included. Primary outcome was respiratory dysfunction at 6 months, defined as at least one among tachypnea at rest, percent predicted 6-min walking distance at 6-min walking test (6MWT) ≤ 70%, pre-post 6MWT difference in Borg score ≥ 1 or a difference between pre- and post-6MWT oxygen saturation ≥ 5%. A nomogram-based multivariable logistic regression model was built to predict primary outcome. Validation relied on 2000-resample bootstrap. The model was compared to one based uniquely on degree of hypoxemia at admission.

**Results:**

Overall, 316 patients were included, of whom 118 (37.3%) showed respiratory dysfunction at 6 months. The nomogram relied on sex, obesity, chronic obstructive pulmonary disease, degree of hypoxemia at admission, and non-invasive ventilation. It was 73.0% (95% confidence interval 67.3–78.4%) accurate in predicting primary outcome and exhibited minimal departure from ideal prediction. Compared to the model including only hypoxemia at admission, the nomogram showed higher accuracy (73.0 vs 59.1%, *P* < 0.001) and greater net-benefit in decision curve analyses. When the model included also respiratory data at 1 month, it yielded better accuracy (78.2 vs. 73.2%) and more favorable net-benefit than the original model.

**Conclusion:**

The newly developed nomograms accurately identify patients at risk of persistent respiratory dysfunction and may help inform clinical priorities.

## Introduction

Coronavirus disease 2019 (COVID-19) has caused substantial morbidity and mortality globally, leading to an unprecedented burden on healthcare systems. Although being a systemic disease, the respiratory system is the front-line of the severe acute respiratory syndrome coronavirus 2 (SARS-CoV-2) infection, with the pattern and extent of lung involvement being the major determinants of clinical outcome (1–3). Mechanisms of lung injury, including diffuse alveolar damage, microvascular thrombosis and immune-mediated damage, may lead to fatal outcome or, if the patient survives the acute phase of disease, to permanent respiratory sequelae (4–7). The post-acute effects of COVID-19 have become an increasing concern and a non-negligible proportion of patients presents lung or respiratory abnormalities at radiological, functional and clinical assessments (8–11), with patients necessitating transfer to the intensive care unit (ICU) being at higher risk of long-term pulmonary complications (11–13). In light of these observations, it is now widely accepted that care for COVID-19 patients may not conclude at the time of hospital discharge (10, 14–16). While long-term clinical monitoring is imperative for ICU patients (17), questions remain on whether prolonged follow-up programs should be extended to all hospitalized patients with COVID-19.

Directing health resources toward post-acute care may be precarious in a time when the large number of acute cases continues to put pressure on health care systems^1^. On the other hand, the global scale of the pandemic suggests that the healthcare needs for COVID-19 survivors will continue to rise^1^. These observations imply that patient prioritization strategies are needed to ensure sustainability of care delivery while guaranteeing the appropriate assistance to most vulnerable patients.

In the current study, we aimed to develop an easy-to-use model to predict reduced respiratory function at 6 months after discharge. Moreover, we examined the discriminant abilities of the model by assessing accuracy, calibration and decision curve analyses (DCA), and compared it with a model based uniquely on the severity of respiratory insufficiency during acute disease. Finally, we tested whether data on respiratory function at 1 month after discharge could increase the prediction accuracy of the newly developed model.

## Materials and Methods

### Study Population

After Institutional Review Board approval (COVID-BioB study, protocol no. 34/int/2020), we prospectively collected data on patients hospitalized for COVID-19 during the first wave of the pandemic at San Raffaele University Hospital in Milan, Italy, and evaluated them at 6 months after hospital discharge at the COVID-19 follow-up outpatient clinic of the same institution. Adult (age ≥ 18 years) individuals, who were not transferred to the intensive care unit (ICU) were included in the present analysis.

COVID-19 was diagnosed based on a positive test result of real-time reverse-transcriptase polymerase chain reaction (RT-PCR) from a nasal swab in the presence of radiological findings of COVID-19 pneumonia. A comprehensive description of the follow-up program and patient assessment protocols are reported elsewhere (10, 16). All participants gave written informed consent. The study was conducted in accordance with the provisions of the Declaration of Helsinki.

### Respiratory Function Assessment and Testing Endpoint

Respiratory function was evaluated using the 6-min walking test (6MWT), as performed according to the guidelines provided by the American Thoracic Society (ATS) (18). A validated reference equation, developed in healthy subjects from seven different countries, was used to derive predictive values of the 6-min walk distance (6MWD) in patients with no history of chronic pulmonary disease (19). A different equation specific for chronic obstructive pulmonary disease (COPD) patients was instead used to calculate COPD-predicted values of the 6MWD (20). Pre- and post-6MWT dyspnea was quantified using Borg scale (18). Respiratory rate (RR) was measured, prior to test initiation, by counting respiratory chest movements over a period of 60 s. A RR > 20 breaths/min defined tachypnea at rest (21).

The endpoint of interest was the ability to predict the presence of decreased respiratory function at 6 months after hospital discharge, defined as the presence of at least one among tachypnea at rest, percent predicted 6MWD ≤ 70%, a difference between pre- and post-6MWT Borg score ≥ 1 or a difference between pre- and post-6MWT oxygen saturation ≥ 5%.

### Variables

Characteristics of the patients, including age, sex, ethnicity, active smoking, pre-existing comorbidities [body mass index (BMI) ≥ 30, arterial hypertension, coronary artery disease, COPD, diabetes mellitus, chronic kidney disease, active neoplasia] and of the disease, including ratio of arterial oxygen partial pressure in mmHg to fractional inspired oxygen expressed as a fraction (PaO_2_/FiO_2_) at hospital admission, length of hospital stay, therapy and non-invasive ventilation (NIV) administration during hospital stay clinical, were used as covariates. The modified Medical Research Council (mMRC) scale for dyspnea (22) and RR (21) were used as a measure of respiratory function at 1 month post-discharge.

### Statistical Analyses

Non-normally distributed continuously-coded variables were expressed as medians and interquartile ranges (IQR). Absolute counts and proportions (%) were reported for categorical variables. The Mann–Whitney and the Chi-squared tests were used to compare medians and proportions, respectively.

Several statistical steps were performed. First, multivariable logistic regression analysis was performed to identify independent predictors of decreased respiratory function at 6 months among all available variables. Then, a logistic regression model was fitted using all identified independent predictors of the outcome. The discriminant properties of the resulting model were examined using ROC-derived area under the curve (AUC). The contribution of the remaining variables to the model was subsequently tested by verifying the AUC of the model when adding each other variable as covariate. Variables proving a benefit > 1% on AUC were included in the final model. Due to non-normal distribution of PaO_2_/FiO_2_, log-transformed values were used in nomogram development. Comparisons between predicted and observed misclassification probabilities for the nomogram were performed. Furthermore, decision curve analysis (DCA) assessed the net-benefit of the nomogram application. Finally, several possible nomogram cut-offs were systematically analyzed (23).

The nomogram was then compared to a model based exclusively on the degree of hypoxia, quantified as PaO_2_/FiO_2_, at hospital admission, using DeLong et al. methodology (24) and DCA analyses. Subgroup analyses were performed on patients who had also undergone the 1-month post-discharge follow-up. Specifically, an additional logistic regression model was fitted using RR and mMRC score at 1 month, in addition to the variables included in the original model. Subsequently, this extended model was compared to the original nomogram, by assessing AUC, calibration plot and DCA. Statistical significance of differences in the resulting AUC values obtained within the same cohort, for, respectively, the original vs. the extended nomogram, was tested according to DeLong et al. methodology (24). For both the original and extended nomograms, 95% confidence interval (CI) of AUC was obtained through 2000-resample bootstrap-based internal validation, which simulated the application of our model to 2000 newly created patient cohorts derived from random resampling of the original population (25, 26). All statistical tests were performed using the R statistical package v.4.0.0 (R Project for Statistical Computing, www.r-project.org). All tests were two sided, with a significance level set at *p* < 0.05.

## Results

A total of 641 COVID-19 survivors who had been previously hospitalized and subsequently discharged from our institution were eligible for follow-up. Of these, 377 underwent the 6-month evaluation between 26 August, 2020 and 16 December, 2020. Sixty-one patients had been transferred to the ICU and were thus excluded. Three-hundred and sixteen COVID-19 survivors were included in the present analyses, and their characteristics are shown in Table 1. Median age was 61.8 (53.9–72.3) years and most patients were males (67.7%). All patients were evaluated after a median time of 187 (180–195) days from hospital discharge.

**Table 1:** Descriptive characteristics of 316 non-critical hospitalized COVID-19 patients, stratified according to the presence of reduced respiratory function at 6 months after hospital discharge.

	**Overall (*n* = 316)**	**Normal respiratory function at 6 months** **(*n* = 198, 62.7%)**	**Reduced respiratory function at 6 months** **(*n* = 118, 37.3%)**	***P***
Age, years	61.8 (53.9–72.3)	61.5 (53.6–70.2)	63.6 (54.7–75.6)	0.09
Length of stay, days	11 (7–18)	11 (7–17)	12 (7–20)	0.35
Time from discharge to 6-month assessment, days	187 (180–195)	187 (180–195)	185 (180–194)	0.25
Female sex	102 (32.3)	50 (25.3)	52 (44.1)	<0.001
Ethnicity				0.65
White	280 (88.6)	175 (88.3)	105 (89)	
Hispanic	28 (8.9)	17 (8.6)	11 (9.3)	
Asian	5 (1.6)	3 (1.5)	2 (1.7)	
Black	3 (0.9)	3 (1.5)	0 (0)	
Active smoking at admission	148 (46.8)	91 (46)	57 (48.3)	0.81
Comorbidities				
HTN	136 (43)	81 (40.9)	55 (46.6)	0.42
Obesity (BMI ≥ 30)	85 (26.9)	40 (20.2)	45 (38.1)	<0.001
CAD	29 (9.2)	15 (7.6)	14 (11.9)	0.28
DM	37 (11.7)	23 (11.6)	14 (11.9)	0.99
COPD	13 (4.1)	2 (1)	11 (9.3)	<0.001
CKD	19 (6)	11 (5.6)	8 (6.8)	0.84
Active neoplasia	12 (3.8)	6 (3)	6 (5.1)	0.53
PaO_2_/FiO_2_ at admission	300 (249–338)	304 (265–339)	287 (216–333)	0.007
Steroid therapy during hospitalization	48 (15.2)	31 (15.7)	17 (14.4)	0.89
NIV administration	89 (28.2)	56 (28.3)	33 (28)	0.99

*Continuous variables were expressed as median (interquartile range), while categorical variables as count (percentage)*.

*BMI, body mass index; HTN, arterial hypertension; CAD, coronary artery disease; DM, diabetes mellitus; COPD, chronic obstructive pulmonary disease; CKD, chronic kidney disease; PaO_2_/FiO_2_, ratio of arterial oxygen partial pressure to fractional inspired oxygen; NIV, non-invasive ventilation*.

Overall, 118 patients (37.3%) had decreased respiratory function, defined as the presence of tachypnea at rest, percent predicted 6MWD ≤ 70%, a difference between pre- and post-6MWT Borg score ≥ 1 or a difference between pre- and post-6MWT oxygen saturation ≥ 5%, at the 6-month follow-up assessment. These patients were more commonly females (44.1 vs. 25.3%, *P* < 0.001) and had a higher BMI than those with normal respiratory function [median (IQR) of 28.3 (24.8–31.9) vs. 27.0 (25.3–29.4), *P* = 0.02]. History of COPD was more frequently recorded in patients with decreased respiratory function (9.3 vs. 1.0%, *P* < 0.001). The two groups did not differ in terms of age, additional comorbidities or steroid treatment during hospital stay.

**Figure 1:**
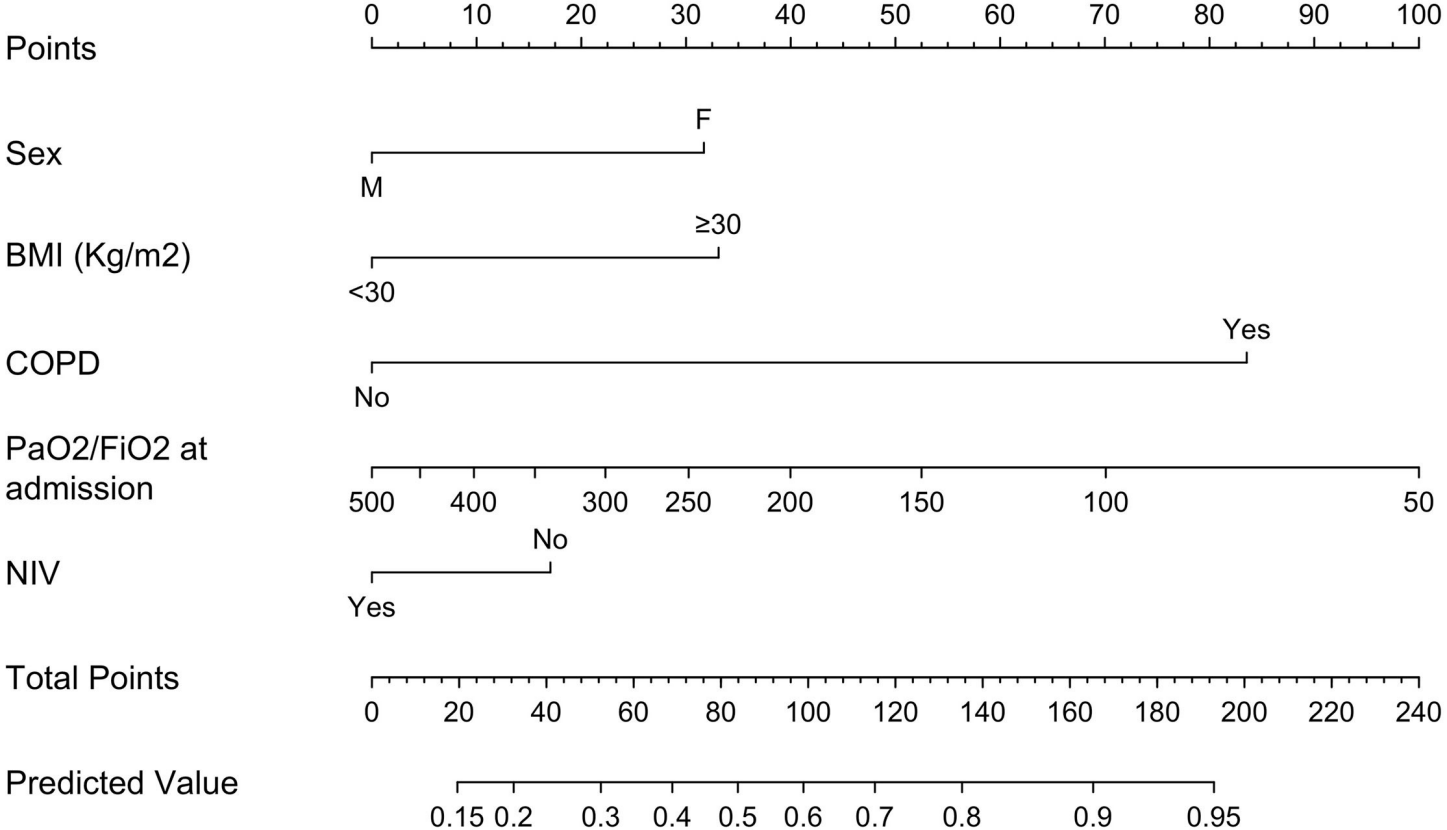
Nomogram predicting respiratory dysfunction at 6 months post-discharge (Original nomogram). BMI, body mass index; COPD, chronic obstructive pulmonary disease; PaO_2_/FiO_2_, ratio of arterial oxygen partial pressure in mmHg to fractional inspired oxygen expressed as a fraction; NIV, non-invasive ventilation.

**Figure 2:**
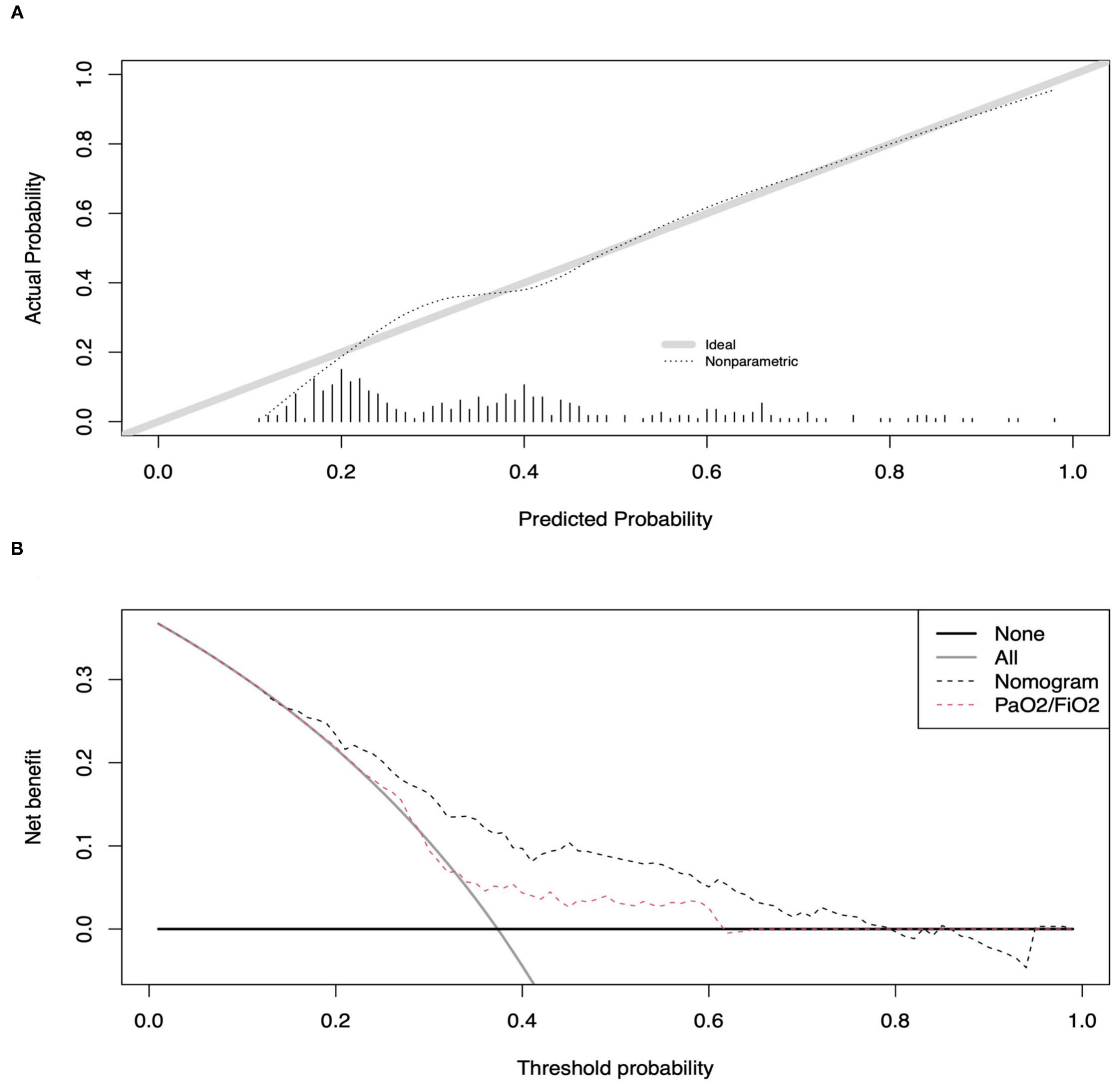
Calibration plot of observed vs. predicted rates of reduced respiratory function at 6 months post-discharge for the newly developed nomogram-based model **(A)**. Decision curve analyses (DCA) demonstrating the net benefit associated with the use of the nomogram on the discrimination of patients with and without reduced respiratory function at 6 months after hospital discharge **(B)**.

The multivariable logistic model used to build the nomogram predicting decreased respiratory function at 6 months relied on sex, baseline BMI ≥ 30, COPD, PaO_2_/FiO_2_ at hospital admission, and NIV administration during hospital stay. All included variables were independent predictors and were used for nomogram development (Supplementary Table 1; Figure 1). An exception was NIV administration that, while not an independent predictor (Supplementary Table 1), increased the accuracy of the model (71.8 vs. 73.0%). Within the overall population, the newly developed nomogram yielded an AUC of 73.0% (95% CI 67.3–78.4%). Comparisons between nomogram-predicted and observed probabilities of decreased respiratory function at 6 months showed minimal departure from ideal prediction (Figure 2A). Moreover, in DCA greater degree of net-benefit was recorded for the nomogram compared to the model relying only on PaO_2_/FiO_2_ at hospital admission (Figure 2B). The nomogram also yielded greater accuracy than the model based only on PaO_2_/FiO_2_ at hospital admission (73.0 vs. 59.1%, *P* < 0.001). Additionally, we tested numbers and proportions of patients with reduced respiratory function at 6 months according to several nomogram cut-offs (Table 2). The systematic analysis of nomogram cut-offs revealed that using, for example, a cut-off of 20, 260 (82.3%) patients would be classified as being at risk of developing decreased respiratory function at 6 months (above nomogram cut-off) and should thus be evaluated at follow-up. On the other hand, 56 (17.7%) should not be evaluated, being below the cut-off. Of these 56 patients, 7 (12.5%) would be misclassified.

**Table 2:** Analyses of novel nomogram cut-offs in 316 non-critical hospitalized COVID-19 patients.

**Nomogram cut-off**	**Patients above nomogram cut-off (%)**	**Number of patients with reduced respiratory function above nomogram cut-off (PPV) (%)**	**Number of patients with reduced respiratory function below nomogram cut-off (1-NPV) (%)**
15	306 (96.8)	117 (38.2)	1 (10)
16	297 (94)	117 (39.4)	1 (5.3)
17	296 (93.7)	117 (39.5)	1 (5)
18	282 (89.2)	116 (41.1)	2 (5.9)
19	272 (86.1)	115 (42.3)	3 (6.8)
20	260 (82.3)	111 (42.7)	7 (12.5)
21	243 (76.9)	105 (43.2)	13 (17.8)
22	230 (72.8)	105 (45.7)	13 (15.1)
23	216 (68.4)	102 (47.2)	16 (16)
24	206 (65.2)	100 (48.5)	18 (16.4)
25	197 (62.3)	97 (49.2)	21 (17.6)
26	191 (60.4)	94 (49.2)	24 (19.2)
27	187 (59.2)	92 (49.2)	26 (20.2)
28	184 (58.2)	91 (49.5)	27 (20.5)
29	183 (57.9)	91 (49.7)	27 (20.3)
30	180 (57)	90 (50)	28 (20.6)
31	175 (55.4)	87 (49.7)	31 (22)
32	169 (53.5)	83 (49.1)	35 (23.8)
33	165 (52.2)	83 (50.3)	35 (23.2)
34	158 (50)	82 (51.9)	36 (22.8)
35	154 (48.7)	81 (52.6)	37 (22.8)

*PPV, Positive predicted value; NPV, negative predictive value*.

**Figure 3:**
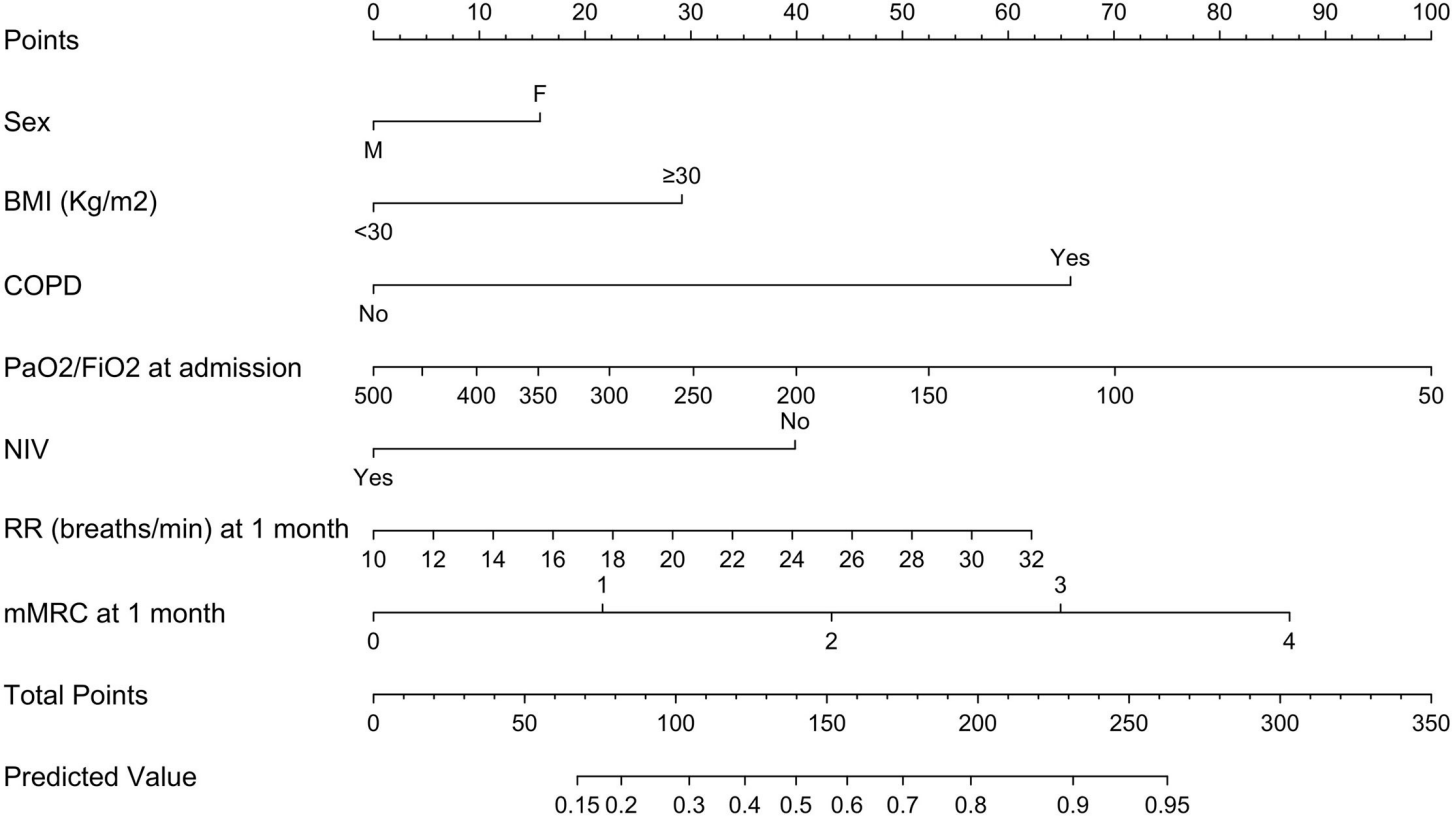
Extended nomogram predicting respiratory dysfunction at 6 months post-discharge, including also respiratory rate (breaths/min) and degree of dyspnea, quantified through the modified Medical Research Council score (mMRC), at 1 month post-discharge. mMRC scores: 0, no dyspnea; 1, mild dyspnea; 2, moderate dyspnea; 3, severe dyspnea; 4, very severe dyspnea. BMI, body mass index; COPD, chronic obstructive pulmonary disease; PaO_2_/FiO_2_, ratio of arterial oxygen partial pressure in mmHg to fractional inspired oxygen expressed as a fraction; NIV, non-invasive ventilation; RR, respiratory rate.

In the subgroup analyses focusing on patients that had also been evaluated at 1-month follow-up, an additional logistic regression model was fitted, relying on the same covariates as the original nomogram (sex, obesity, COPD, PaO_2_/FiO_2_ at hospital admission and NIV administration) in addition to RR and mMRC for dyspnea score at 1 month (Figure 3). Overall, 220 patients assessed after a median time of 34 (25–40) days after hospital discharge were included. Of these, 77 (35%) had reduced respiratory function at 6 months. Median RR (breaths/min) at 1 month was 20 (16–21) in patients with reduced respiratory function at 6 months and 16 (15–20) in patients without (*P* = 0.002). Similarly, patients with reduced 6-month respiratory function had higher degrees of dyspnea according to mMRC score compared to patients without (*P* < 0.001). For example, severe and very severe dyspnea were reported in, respectively, 11 (14.3%) and 8 (10.4%) patients with reduced respiratory function at 6 months and in, respectively, 4 (2.8%) and 1 (0.7%) patients without.

The extended nomogram yielded an AUC of 78.2% (95% CI 72.0–84.1%) vs. 73.2 (95% CI 66.4–79.8%) for the original nomogram in the sub-cohort (*P* = 0.03). Comparisons between nomogram-predicted and observed probabilities of decreased respiratory function at 6 months showed a good calibration for both the original and the extended nomograms (Supplementary Figures 1A,B). Moreover, in DCA a greater degree of net-benefit was recorded for the extended nomogram across all threshold probabilities, with the exception of the interval between 0.51 and 0.64, where the net-benefit was greater for the original nomogram (Supplementary Figure 1C). Numbers and proportions of patients with reduced respiratory function at 6 months according to several cut-offs are reported in Supplementary Table 2.

## Discussion

In our cohort of non-critical COVID-19 survivors, more than one third of patients had respiratory dysfunction at 6-month follow-up. Here, we developed an accurate and easy-to-use model to discriminate patients deserving prolonged monitoring due to an increased risk of respiratory *sequelae*.

Respiratory function and lung abnormalities including dyspnea, reduced exercise tolerance, decreased diffusion lung capacity (DL_CO_) and parenchymal changes frequently develop during acute COVID-19 and persist after patients discharge (9, 11, 15, 27). This evidence has led to the increasing recognition of post-discharge care of COVID-19 survivors as a clinical and research priority. Prolonged monitoring for ICU patients is considered appropriate (11). However, respiratory sequelae also occur in hospitalized patients who did not need ICU transfer. Data on the prevalence of this event are scarce and consequently it is unclear whether these patients should be included in long-term follow-up programs (28).

To address this unmet need, we developed a model, in the form of a nomogram, for the identification of patients with reduced respiratory function at 6 months after hospital discharge. Exercise tolerance and respiratory rate at rest are informative clinical surrogates of respiratory function (21, 29–32), and have been suggested as tools for respiratory follow-up in post-acute COVID-19 patients (16, 17). Indeed, the distance walked during the 6MWT was shown to be closely linked to disease severity in diffuse parenchymal lung diseases (33) and to faithfully mirror respiratory function in interstitial lung diseases (34). Similarly, RR was found to be a valid and clinically useful indicator of respiratory dysfunction in both acute and chronic lung disorders (35, 36).

Several reference equations exist to define predicted values of normality for 6MWD (19, 37–41). The one we used, by Casanova et al. was developed in adult patients from seven countries and, differently from other equations (37, 40, 41), takes into account the intensity of effort during the test, which may otherwise bias the results (19).

The nomogram, which rested on sex, obesity, COPD, PaO_2_/FiO_2_ at hospital admission, and NIV administration during hospital stay, yielded an AUC of 73.0%, above the ideal accuracy threshold of 70%. Several other testing benchmarks were applied to this novel nomogram-based model, namely calibration, DCA, and detailed analysis of misclassified patients according to specific cut-offs. Calibration analyses showed only minimal departures of the model from ideal predictions. Importantly, the newly developed model demonstrated statistically significant superiority of performance compared to the one based uniquely on PaO_2_/FiO_2_ at hospital admission (AUC 59.1%), as reflected also by the greater net-benefit in DCA analyses. Indeed, PaO_2_/FiO_2_ is a more objective surrogate marker of disease severity than the oxygenation or ventilation strategy used, as it directly reflects the oxygen need (42, 43), without being influenced by subjective clinical judgment. The ability of disease severity to predict decreased exercise capacity at follow-up has been previously reported (11). However, we here demonstrate that disease severity, although extremely important, may not be sufficient to accurately predict the risk of long-term respiratory sequelae. Indeed, in line with a previous study reporting an increased risk of reduced DL_CO_ at 4 months in female patients (9, 44), female sex importantly contributed to nomogram accuracy. Similarly, in accordance with our model, COPD was shown to increase the risk of post-acute lung function impairment (9).

The use of COPD-specific reference equations in our study minimized the risk of bias related to the potential pre-existing reduction in exercise capacity in COPD patients. Age did not emerge as an independent predictor of decreased respiratory function at 6 months and was thus not included in the nomogram. A higher in-hospital mortality rate was recorded in the elderly (3, 45), probably due to a greater baseline comorbidity burden. However, in survivors, the ability to revert the previous health state may align with that of younger patients. We have also found that NIV administration, although not being an independent predictor per se, improved the nomogram accuracy when added as covariate. Therefore, it is tempting to speculate that in non-critical COVID-19 patients NIV contributes to limit acute lung damage, thus protecting from long-term respiratory sequelae, consistent with the evidence of its efficacy during acute disease (46).

Subgroup analyses revealed that by adding respiratory measures (RR and degree of dyspnea) at 1 month post-discharge to the original nomogram, the prediction accuracy of the model improved significantly (AUC 78.2%). Thus, early clinical assessment of respiratory function after patient discharge might aid in the prediction of those patients warranting more prolonged medical care.

Our study has limitations. First, not all patients discharged from our Institution underwent follow-up assessment, due to several reasons including reluctance to attend extra-visits, perception of full recovery, inability to reach the outpatient clinic, death due to COVID-19 or other causes, etc. However, the homogeneity of standards of care for patient monitoring and the uniform healthcare access of the cohort minimized the risk of ascertainment bias. Second, only patients who were evaluated at both the 1- and 6-month visits were included in subgroup analyses. Third, multiple factors such as myopathy, depression or neuropathy may have influenced exercise performance. However, the 6MWT captures the global functional capacity of a patient (30), which, independent of contributing elements to exercise limitation, imparts important prognostic implications and should guide clinical decision-making. Moreover, data on pre-existing respiratory diseases other than COPD were not available. Also, few patients with severe disease who had not been admitted to the ICU due to non-clinical reasons (refusal, limited ICU capacity, etc.) may have been included. However, the proportion of these patients is supposed to be very low, considering the high mortality rate of patients who received less intensive care than needed. Finally, the relatively small sample size and the lack of an external validation cohort may hamper result generalizability, although internal validation confirmed model accuracy.

## Conclusion

We developed the first nomogram-based model to identify patients at higher risk of reduced respiratory function at 6 months after hospital discharge. The nomogram displayed good performance, based on AUC, calibration and DCA. Moreover, the flexible format of nomogram-based predictions allows for the use of the cut-off that best suits the clinical need and available resources, balancing the number of patients classified as at risk of persistently reduced respiratory function vs. the proportion, within those patients, that are misclassified. Thus, the developed nomogram is an evidence-based, easy-to-use tool that could be readily implemented in clinical practice to prioritize care delivery for non-critical COVID-19 patients, with the ultimate aim of preserving valuable resources while minimizing the long-term effects of this devastating disorder.

## Data Availability Statement

The raw data supporting the conclusions of this article will be made available by the authors, without undue reservation.

## Ethics Statement

The study was reviewed and approved by Institutional Review Board of San Raffaele Hospital. The patients/participants provided their written informed consent to participate in this study.

## Author Contributions

RD: conception and design, acquisition of data, analysis and interpretation of data, statistical analyses, and drafting of manuscript. CM, EC, GV, SM, MM, MC, SD, NC, and MF: acquisition of data and critical revision of the manuscript. LN: analysis and interpretation of data, statistical analyses, and critical revision of the manuscript. CC, FB, and FC: critical revision of the manuscript and supervision. PR-Q: conception and design, analysis and interpretation of data, drafting of the manuscript, and supervision. All authors contributed to manuscript revision, read, and approved the submitted version.

## Funding

This work was funded by Ministero della Salute, Italy; COVID-19 donations.

## Conflict of Interest

The authors declare that the research was conducted in the absence of any commercial or financial relationships that could be construed as a potential conflict of interest.

## Publisher's Note

All claims expressed in this article are solely those of the authors and do not necessarily represent those of their affiliated organizations, or those of the publisher, the editors and the reviewers. Any product that may be evaluated in this article, or claim that may be made by its manufacturer, is not guaranteed or endorsed by the publisher.

## Footnotes

^1^Available online at: https://www.who.int/emergencies/diseases/novel-coronavirus-2019 (accessed April 5, 2021).

## Supplementary Material

Supplementary Figure 1 Calibration plot of observed vs. predicted rates of reduced respiratory function at 6 months post-discharge for the original nomogram-based model **(A)**. Calibration plot of observed vs. predicted rates of reduced respiratory function at 6 months post-discharge for the extended nomogram-based model **(B)**. Decision curve analyses (DCA) demonstrating the net benefit associated with the use of the original nomogram vs. the extended nomogram on the discrimination of patients with and without reduced respiratory function at 6 months after hospital discharge **(C)**.

Supplementary Table 1 Selection criteria for covariates included in the nomogram.

Supplementary Table 2 Analyses of the extended nomogram cutoffs in 220 non-critical hospitalized COVID-19 patients.

## Abbreviations

COVID-19: Coronavirus Disease 2019
SARS-CoV-2: Severe acute respiratory syndrome coronavirus 2
DCA: decision curve analyses
ICU: intensive care unit
RT-PCR: real-time reverse-transcriptase polymerase chain reaction
6MWT: 6-min walking test
6MWD: 6-min walk distance
COPD: chronic obstructive pulmonary disease
RR: respiratory rate
BMI: body mass index
PaO_2_/FiO_2_: ratio of arterial oxygen partial pressure in mmHg to fractional inspired oxygen expressed as a fraction
NIV: non-invasive ventilation
mMRC: modified Medical Research Council
IQR: interquartile range
AUC: ROC-derived area under the curve.
