# Supplementary figures and images for: A Nomogram-Based Model to Predict Respiratory Dysfunction at 6 Months in Non-Critical COVID-19 Survivors
Source: Front Med (Lausanne). 2022 Feb 23;9:781410. doi: 10.3389/fmed.2022.781410 (PMC8904385; doi:10.3389/fmed.2022.781410)

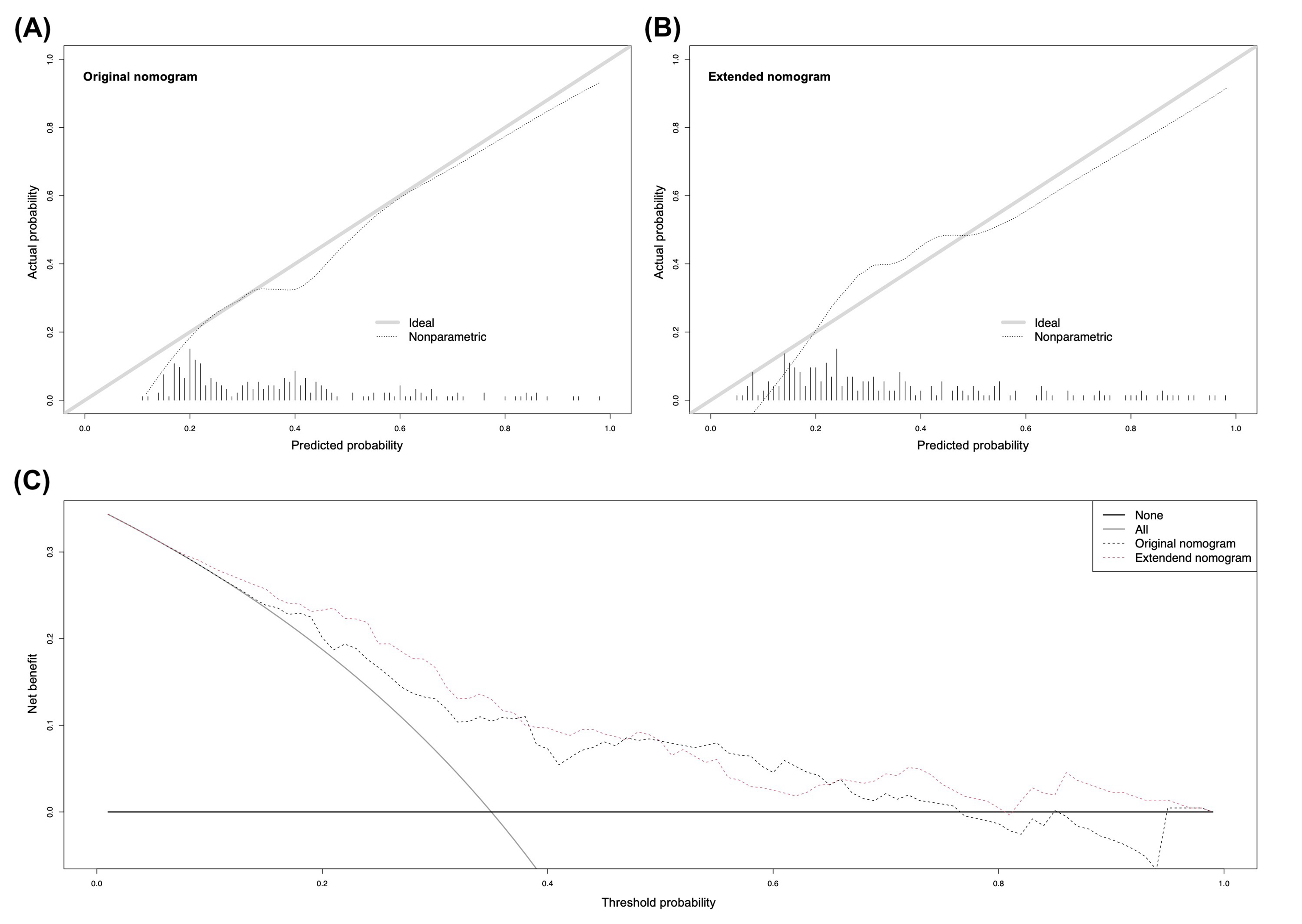

Supplement: Supplementary Figure 1 — Calibration plot of observed vs. predicted rates of reduced respiratory function at 6 months post-discharge for the original nomogram-based model (A). Calibration plot of observed vs. predicted rates of reduced respiratory function at 6 months post-discharge for the extended nomogram-based model (B). Decision curve analyses (DCA) demonstrating the net benefit associated with the use of the original nomogram vs. the extended nomogram on the discrimination of patients with and without reduced respiratory function at 6 months after hospital discharge (C). [file Image_1.JPEG]
