# Supplementary material for: A Nomogram-Based Model to Predict Respiratory Dysfunction at 6 Months in Non-Critical COVID-19 Survivors
Source: Front Med (Lausanne). 2022 Feb 23;9:781410. doi: 10.3389/fmed.2022.781410 (PMC8904385; doi:10.3389/fmed.2022.781410)
Supplement: Supplementary Table 1 — Selection criteria for covariates included in the nomogram. [file Table_1.docx]

|  | **Step 1. Multivariable logistic regression** | | **Step 2. Contribution to AUC of the model based on independent predictors** |
| --- | --- | --- | --- |
| **Variable** | **OR** | **P** | **AUC_2_ – AUC_1_** |
| Age (years) | 1.01 | .70 | + 0.2 |
| Female sex | 2.37 | **.003** | **–** |
| Ethnicity | 1.01 | .97 | 0 |
| Active smoking at admission | 1.00 | .99 | + 0.1 |
| Obesity (BMI ≥30) | 2.71 | **<.001** | **–** |
| HTN | 0.87 | .64 | 0 |
| CAD | 1.48 | .45 | + 0.4 |
| DM | 0.61 | .26 | + 0.3 |
| COPD | 7.88 | **.01** | **–** |
| CKD | 1.49 | .47 | - 0.2 |
| Active neoplasia | 1.72 | .42 | + 0.4 |
| Steroid therapy | 0.91 | .80 | + 0.2 |
| PaO_2_/FiO_2_ at admission | 0.98 | **.001** | **–** |
| NIV administration | 0.58 | .15 | **+ 1.2%** |
| Length of stay (days) | 1.01 | .66 | 0 |

**Supplementary Table 1.** Selection criteria for covariates included in the nomogram.

OR, odds ratio. AUC, area under the receiver operating characteristic curve. BMI, body mass index. HTN, arterial hypertension. CAD, coronary artery disease. DM, diabetes mellitus. COPD, chronic obstructive pulmonary disease. CKD, chronic kidney disease. PaO_2_/FiO_2_, ratio of arterial oxygen partial pressure to fractional inspired oxygen NIV, non-invasive ventilation.

AUC_1_: nomogram resting on all independent predictor variables. AUC_2_: nomogram resting on all independent predictor variables as well as the indicated variable.
