# Supplementary material for: A Nomogram-Based Model to Predict Respiratory Dysfunction at 6 Months in Non-Critical COVID-19 Survivors
Source: Front Med (Lausanne). 2022 Feb 23;9:781410. doi: 10.3389/fmed.2022.781410 (PMC8904385; doi:10.3389/fmed.2022.781410)
Supplement: Supplementary Table 2 — Analyses of the extended nomogram cutoffs in 220 non-critical hospitalized COVID-19 patients. [file Table_2.docx]

**Supplementary table 2.** Analyses of the extended nomogram cutoffs in 220 non-critical hospitalized COVID-19 patients.

| **Nomogram cut-off** | **Patients above nomogram cut-off (%)** | **Number of patients with reduced respiratory function above nomogram cut-off [PPV] (%)** | **Number of patients with reduced respiratory function below nomogram cut-off [1-NPV] (%)** |
| --- | --- | --- | --- |
| 10 | 301 (95.3) | 110 (36.5) | 8 (53.3) |
| 11 | 297 (94) | 109 (36.7) | 9 (47.4) |
| 12 | 291 (92.1) | 107 (36.8) | 11 (44) |
| 13 | 286 (90.5) | 104 (36.4) | 14 (46.7) |
| 14 | 280 (88.6) | 103 (36.8) | 15 (41.7) |
| 15 | 268 (84.8) | 99 (36.9) | 19 (39.6) |
| 16 | 256 (81) | 92 (35.9) | 26 (43.3) |
| 17 | 246 (77.8) | 88 (35.8) | 30 (42.9) |
| 18 | 236 (74.7) | 82 (34.7) | 36 (45) |
| 19 | 233 (73.7) | 80 (34.3) | 38 (45.8) |
| 20 | 223 (70.6) | 74 (33.2) | 44 (47.3) |
| 21 | 212 (67.1) | 71 (33.5) | 47 (45.2) |
| 22 | 207 (65.5) | 68 (32.9) | 50 (45.9) |
| 23 | 195 (61.7) | 64 (32.8) | 54 (44.6) |
| 24 | 188 (59.5) | 61 (32.4) | 57 (44.5) |
| 25 | 172 (54.4) | 56 (32.6) | 62 (43.1) |
| 26 | 169 (53.5) | 55 (32.5) | 63 (42.9) |
| 27 | 162 (51.3) | 53 (32.7) | 65 (42.2) |
| 28 | 156 (49.4) | 51 (32.7) | 67 (41.9) |
| 29 | 153 (48.4) | 51 (33.3) | 67 (41.1) |
| 30 | 146 (46.2) | 47 (32.2) | 71 (41.8) |
| 31 | 140 (44.3) | 43 (30.7) | 75 (42.6) |
| 32 | 132 (41.8) | 41 (31.1) | 77 (41.8) |
| 33 | 128 (40.5) | 41 (32) | 77 (41) |
| 34 | 120 (38) | 37 (30.8) | 81 (41.3) |
| 35 | 117 (37) | 36 (30.8) | 82 (41.2) |

PPV, Positive predicted value. NPV, negative predictive value.
